# Differential Patterns of Prefrontal MEG Activation during Verbal & Visual Encoding and Retrieval

**DOI:** 10.1371/journal.pone.0082936

**Published:** 2013-12-16

**Authors:** Garreth Prendergast, Eve Limbrick-Oldfield, Ed Ingamells, Susan Gathercole, Alan Baddeley, Gary G. R. Green

**Affiliations:** 1 York NeuroImaging Centre, University of York, York, United Kingdom; 2 Department of Psychology, University of Cambridge, Cambridge, United Kingdom; 3 Department of Psychology, University of York, York, United Kingdom; 4 Medical Research Council Cognition and Brain Sciences Unit, University of Cambridge, Cambridge, United Kingdom; University College of London - Institute of Neurology, United Kingdom

## Abstract

The spatiotemporal profile of activation of the prefrontal cortex in verbal and non-verbal recognition memory was examined using magnetoencephalography (MEG). Sixteen neurologically healthy right-handed participants were scanned whilst carrying out a modified version of the Doors and People Test of recognition memory. A pattern of significant prefrontal activity was found for non-verbal and verbal encoding and recognition. During the encoding, verbal stimuli activated an area in the left ventromedial prefrontal cortex, and non-verbal stimuli activated an area in the right. A region in the left dorsolateral prefrontal cortex also showed significant activation during the encoding of non-verbal stimuli. Both verbal and non-verbal stimuli significantly activated an area in the right dorsomedial prefrontal cortex and the right anterior prefrontal cortex during successful recognition, however these areas showed temporally distinct activation dependent on material, with non-verbal showing activation earlier than verbal stimuli. Additionally, non-verbal material activated an area in the left anterior prefrontal cortex during recognition. These findings suggest a material-specific laterality in the ventromedial prefrontal cortex during encoding for verbal and non-verbal but also support the HERA model for verbal material. The discovery of two process dependent areas during recognition that showed patterns of temporal activation dependent on material demonstrates the need for the application of more temporally sensitive techniques to the involvement of the prefrontal cortex in recognition memory.

## Introduction

Links between episodic memory and the frontal lobes are well established: frontal lesions result in episodic memory deficits [1], [2], and neuroimaging studies have consistently demonstrated prefrontal cortex (PFC) activations in episodic memory tasks [3], [4], [5], [6]. However, the precise characteristics and lateralisation of frontal activity during episodic memory tasks is dependent upon multiple factors that have not yet been fully characterized. Evidence to date indicates that lateralisation of PFC activity is dependent both on the type of material used [5], [7], [8] and on whether encoding or retrieval processes are involved [3], [4].

The purpose of this study was to provide a systematic investigation of lateralisation in the PFC for episodic memory processing and to uncover to what extent this is a dynamic process. The existing models are static and simplistic, with information transfer between spatially distinct nodes of activity, but it may be the case that this network of activity changes during the process of encoding and retrieving information and an accurate model of the neural underpinnings may have specific time windows of importance. The experiments described utilised the Doors and People Test [9], which is a popular neuropsychological test designed to invoke verbal and visual episodic memory. Previous work has demonstrated the utility of the test in dissociating verbal and visual memory systems in unilateral temporal lobectomy patients [10]. Right temporal lobectomy patients were impaired on the Doors test of visual recognition memory, with relative preservation of scores in the People test of verbal recognition memory. Patients with a left temporal lobectomy displayed the opposite pattern, thus demonstrating that the test is appropriate to elucidate the lateralisation of function within the PFC as a function of the material to be remembered. There is value in using a standard, widely known stimulus set to investigate the neural correlates of encoding and retrieving information, as there exists a behavioural literature specific to this task which aids the interpretation of the observed data.

lateralisation of verbal and visual memory systems has been demonstrated within the frontal cortex in patient groups where unilateral regions of the cortex were surgically removed [2]. Patients with left mid-lateral frontal regions removed exhibited deficits in verbal recency judgements, whereas those with right frontal regions removed had significant deficits in pictorial recency tasks. Verbal and visual memory deficits in self-ordering tasks for have also been demonstrated in patients with left frontal excisions, but only visual deficits for right frontal excision patients [1]. However, it is also reported that verbal recognition memory deficits are seen in patients with right frontal lobe damage, compared to left damage patients and matched controls [11].

The use of functional neuroimaging has led to the development of two competing theories regarding the role of the pre-frontal cortex in episodic memory. The first theory proposes material-specific laterality within the PFC, with functional magnetic resonance imaging (fMRI) showing PFC activation modulated by material type [5], [7]. Using block design fMRI and a basic recognition paradigm to study the encoding of words, nameable line-drawn objects and unfamiliar faces it was shown that encoding of words produced left dorsolateral PFC (DLPFC) activation whereas unfamiliar faces activated the right DLPFC, with nameable line drawings activating the DLPFC bilaterally [5]. These findings were replicated [7], and it was suggested that laterality was modulated by the degree to which the material could be verbally recoded. A corresponding DLPFC material-laterality effect has also been demonstrated in the auditory domain [12]. Further fMRI studies extend these findings by showing a material-specific laterality effect over the retrieval phase of episodic memory for words and textures [8], as well as words and unfamiliar faces [13].

The second theory describing the role of the PFC in episodic memory is the hemispheric encoding-retrieval asymmetry (HERA) model proposed by Tulving et al [3]. This model suggests that the left PFC is predominantly involved in the encoding process of memory, whereas the retrieval process is carried out in the right PFC. The original model was proposed using purely verbal material. However, a subsequent review of PET studies [4] demonstrated that the model could be extended to non-verbal tasks. The HERA model received heavy criticism over recent years due to the aforementioned studies finding differential PFC activation dependent on material type. However McDermott et al, in addition to finding material laterality in the DLPFC, found an area in the right frontal polar cortex that showed significantly greater activation for retrieval than encoding and this was independent of material type [13]. This process dependent area fits the HERA model. Habib et al.[14] suggest there are different asymmetries in the PFC, resulting in the conflicting findings. They argue that HERA is only relevant in a direct comparison of encoding and retrieval conditions and that if a passive baseline is used for comparison, HERA activation is not revealed. More recent support for the HERA model has come from electroencephalography (EEG) recordings which measured the coherence of cortical signals measured during an episodic memory task [15]. Activity in the Gamma band (30–45 Hz) originating from fronto-parietal areas was found to be left lateralised for encoding and right lateralised for retrieval of non-verbal visuo-spatial scenes. The encoding and retrieval of complex scenes has also been studied whilst repetitive transcranial magnetic stimulation (rTMS) was applied to the PFC. Stimulation of the left DLPFC significantly disrupted encoding whereas stimulation to the right disrupted retrieval [16]. This finding provides support for the HERA model. However, a direct comparison of verbal and non-verbal material demonstrates that verbal encoding was disrupted by application of TMS to the left PFC and non-verbal by application to the right PFC [17]. Differing results have also been seen in other rTMS studies (e.g. [18], [19], [20]).

The use of EEG and magnetoencephalography (MEG) allows not only the location of the mechanisms involved in memory to be determined, but also provides information regarding the timings and oscillatory changes in power that are associated with such networks. These techniques allows the characterisation of prefrontal memory function was a dynamic network, and this is an approach that thus far is not extensively covered in the literature. For example it has been reliably demonstrated with MEG the involvement of theta and gamma frequency bands in episodic memory during encoding for successfully recognised material [21]. Additionally, intracranial EEG has been used to establish the involvement of gamma activity, and theta activity during successful word encoding [22]. To date, MEG studies investigating episodic memory have largely concentrated on the medial temporal lobe (MTL) (e.g. [23], [24]). The majority of studies in MEG use dipolar techniques to perform the inverse modelling. Spatial filters are an effective tool in analysing networks involved in memory function, although in their study they focused on levels of coherence in visual areas [21]. It is inherently difficult to use spatial filters to analysis MEG signals generated by the hippocampal structures during a memory task [25]. However, spatial filters have not been previously used to focus the analysis on PFC structures. The difference between the current study and previous work [21] is that they focused on the visual activity and how the transfer of information in these cortical regions is modulated by task and stimulus type. The current study aims to focus on the PFC, and to begin understanding the neural dynamics and oscillatory signatures found in these frontal regions during the encoding and subsequent retrieval of different stimulus types. Many neuroimaging studies investigating memory function do not explicitly investigate the prefrontal regions. A common approach is to compare changes in activity when processing old and new items, however this paradigm typically recruits parietal/temporoparietal regions and does not clearly show prefrontal activity.

The question of PFC laterality as a function of material type or process has not yet been addressed using MEG, and is the main focus of the present study. The temporal sensitivity of MEG makes it a highly appropriate method to study this issue. Furthermore, a possible cause of the conflicting neuroimaging evidence to date on PFC laterality may be the lack of temporal resolution of fMRI and PET. This study represents a first step in characterising the temporal dynamics of the prefrontal network during memory function. The starting point for this work is the use of a known, widely used neuropsychological test of verbal and non-verbal memory. The key aims of the study will be to learn whether MEG and a inverse modelling is an approach well-suited to the study of the PFC. The two models described in the literature give clear predictions regarding which stimulus-hemisphere couplings will show the greater neuronal activity during encoding and retrieval and it is of interest to observe whether there is strong evidence for a single one of these models or whether there is a model which better accounts for the observed data in which aspects of both existing models appear to be accurate.

In summary, as the previous discussion demonstrates, both MEG and EEG are suitable methodologies for studying oscillatory activity in the gamma and theta ranges. Theta oscillations have been found to be important in memory tasks and have been found to be localised to the frontal cortices [26], [27]. The current study represents a first attempt to use inverse modelling to systematically investigate the laterality of theta oscillations in the prefrontal cortex. Such an approach is predicted to be of interest as MEG inverse modelling, the prefrontal cortices and theta and gamma activity have all been the focus of previous studies, but this is the first time they have been used together to answer the specific question of interest.

## Methods

### Participants

Sixteen neurologically healthy participants with a mean age of 22 years (Range 19–31; 9 females) were recruited from the University of York student population and paid for their participation. All participants were native English speakers, had normal or corrected to normal vision, no history of language impairment and were right handed, as assessed by the Annett Hand Preference Questionnaire [28]. The study was approved by the York Neuroimaging Centre Ethics Committee and written informed consent was obtained from each participant.

### Design and Materials

The Doors and Names recognition tests used were adapted from the Doors and People test battery [9]. The original protocol of the battery was modified in several respects to fit with scanning constraints and requirements. First, the response time in the recognition phase was restricted to three-seconds. Second, the stimuli were presented serially in the recognition phase,, as opposed to the original 2-by-2 array. Thus the task required a yes/no response to each picture depending on whether the picture had been seen in the encoding phase. The use of a serial presentation procedure was necessary to reduce the artefacts produced by excessive eye movement and to decrease the test difficulty in order to accommodate the reduced viewing time.

### Stimuli

**Figure 1:**
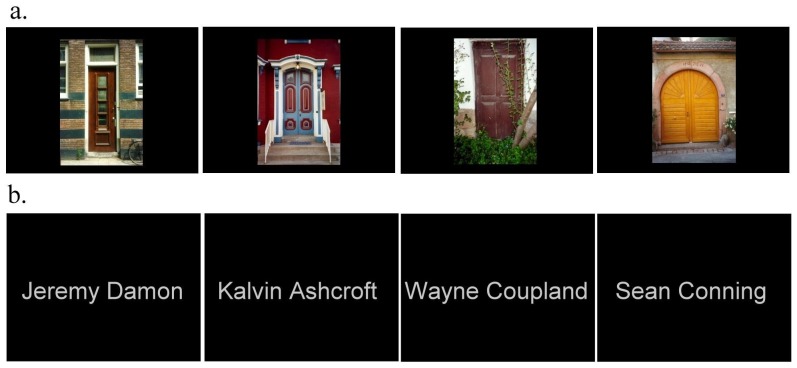
Examples taken from the stimulus set (not to scale). (a) Four examples of the Door stimuli. (b) Four examples of the Name stimuli.

For the Doors recognition test, 128 real-life photographs of Doors were selected from the original corpus of 2500. The stimuli were full colour with a resolution of 230×350 pixels subtending a visual angle of 8.0×13.1° and were easily distinguishable from each other. They were presented centrally against a black background of 768×1000 pixels. For the verbal recognition test, 128 Names were created. Each had a forename and surname, and no part of the name was used more than once. Names were created with specific attention paid to reducing potential visual imagery that may be used to assist encoding or recognition. Each Name was presented centrally in white sans text, capitalised, on a black background of 768×1000 pixels subtending a visual angle of between 4.5×0.7° and 8.0×0.7°. All stimuli were presented on a suspended 1.5×1.2 m rear projection screen at a distance of 1 m using a Dukane 8942 ImagePro 4500 lumens LCD projector. Examples from the stimulus set are shown in figure 1.

### Task

**Figure 2:**
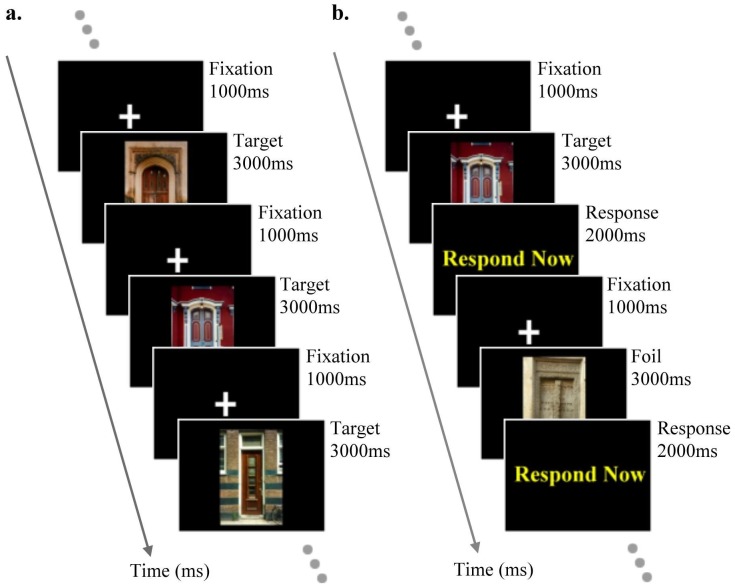
Experimental paradigm. (a) Encoding paradigm – 12 target stimuli preceded by baseline fixation cross. (b) Recognition paradigm – 12 target and 4 foil stimuli preceded by a baseline fixation cross, and followed by a response screen.

Of the 128 stimuli for each test, 96 were randomly chosen as targets, and 32 as foils. This ratio of targets to foils has previously been shown to provide the most reliable and consistent MEG activation profiles (Breier et al., 2000). Both sets of stimuli were split into 8 blocks, each comprising 12 target items and 4 foil items. The encoding phase consisted of the serial presentation of the 12 target stimuli; each stimulus was presented for 3000ms and was preceded by a central fixation cross for 1000ms. The order of presentation was randomised within the blocks. Before the encoding phase started the participant was instructed to remember the stimuli that followed. The recognition phase immediately followed the encoding phase. The same 12 targets from the encoding phase were presented again, with 4 foils, in a randomised order for 3000ms each. Each stimulus was preceded by a 1000ms fixation cross, and followed by a 2000ms screen on which the words ‘*Respond Now*’ were centrally presented. During this response screen the participant was required to indicate whether the preceding stimulus had been seen before. Participants indicated their decision on a response pad, using the left index finger to indicate a target item (old) and the left middle finger to indicate a foil (new). See figure 2 for a representation of the experimental paradigm during the encoding and recognition phases.

**Figure 3:**
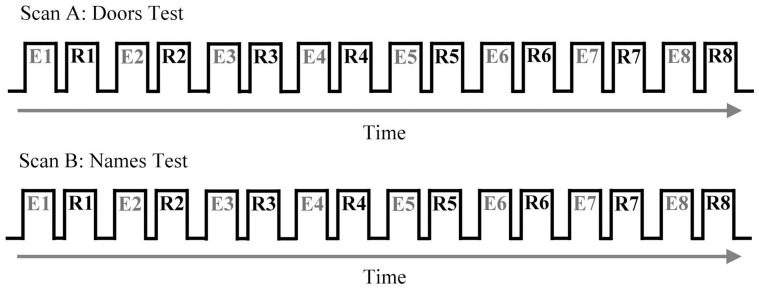
Experimental design: E = Encoding; R = Recognition. Representation of the consecutive presentation of 8 stimuli blocks within one scan.

There were eight blocks of stimuli consisting of an encoding and a recognition phase for each test. The experimental session was split into two 20-minute scans; one for the Doors test and one for the Names test. The order of the scans was counterbalanced between participants to reduce potential practice and fatigue effects. The participant was provided with a short break between the scans. The structure of the scanning sessions is shown in Figure 3.

Each participant received full instructions and carried out a short practice on both tasks before being placed in the scanner. These contained six items in encoding and eight items in recognition (six targets, two foils).

During the design of the paradigm behavioural testing was carried out to ensure task difficulty was comparable across tests. A dependent *t*-test revealed no significant difference in task performance between Door trials (M =  82.38, SE =  4.19) and Name trials (M =  78.88, SE =  3.66, *t*(7) =  1.13.). The tests were designed to ensure performance was good so that there would be enough successful memory trials to be analysed.

### Data Acquisition

Continuous recordings were made throughout stimulus presentation using a 248 whole-head squid magnetometer system (4D Neuroimaging, San Diego, CA) at the York Neuroimaging Centre. A sampling rate of 678.17 Hz was used with a bandwidth of 200 Hz. MEG data for each participant were co-registered to a T1 weighted structural 3T MRI image acquired with a voxel size of 1.13×1.13×1.0 mm (GE Systems). A 3D digitiser (Polhemus Fastrak, Colchester, VT) was used to obtain a representation of the individual head-shape within the MEG scanner and this was then mapped onto the MR image using a technique of distance minimisation adapted from [29].

### Data Analysis

Epochs corresponding to recognition trials in which the participant correctly identified a target (a hit) were further analysed. All misses (incorrectly identifying a target as new) and foil trials were not analysed. Only the encoding epochs that corresponding to correctly identified targets were further analysed, with all encoding targets not subsequently identified correctly omitted from the analysis. Thus there were four conditions to analyse; successful Door encoding trials, successful Name encoding trials, successful Door recognition trials and successful Name recognition trials. There is interest in analysing foil stimuli and stimuli that were not successfully encoded, but such an analysis would require a large number of trials and therefore the approach taken for this initial study was to ensure that there was sufficient signal-to-noise to observed stimulus-related changes in the prefrontal cortex. This preliminary study could then be used to calculate the number of trials needed to characterise the network and a protocol developed to ensure that enough trials and foils were present for the analysis.

### Sensor space analysis

Phase-locked responses were computed in by averaging across repeated trials. A group of 23 sensors covering the frontal lobe were then averaged and a time-frequency decomposition of this average waveform was computed. The analysis was performed for each participant and the resulting event-related field representations were used to inform the selection of filter-band and window parameters for the inverse modelling.

### Inverse modelling

Two contiguous analysis windows were used to provide not only a volumetric estimation of active brain regions but also some coarse information related to timing. For encoding trials, the first analysis window started at 100 ms post-stimulus onset and extended to 600 ms post-stimulus. The second window covered 600–1100 ms post-stimulus. For retrieval trials the windows were moved to be later in time as inspection of the sensor activity revealed the responses were delayed and so they covered 300–800 ms and 800–1300 ms post-stimulus. Each of these active windows was 500 ms in duration and was contrasted against a window of passive activity taken from the inter-stimulus interval (600–100 ms pre-stimulus). The use of a 500-ms analysis window allows sufficient frequency resolution to accurately characterise cortical oscillatory activity at low frequencies. The use of two contiguous windows provides some information regarding how this activity is modulated over time.

Source localisation was performed using a vectorised beamformer based on a linearly constrained minimum variance spatial filter [30], also described as a Type I beamformer [31]. A grid of points was placed throughout the cortical volume (with a spatial resolution of 5 mm for the work described) and at each point an independent spatial filter was constructed. Derivations of the specific implementation used are given in a previous paper [32]. Typically a volumetric map of power is produced for a time window and frequency band of interest, an “active” window, and also a period thought to represent a baseline comparison, a so-called “passive” window. These maps are then compared voxel-by-voxel using a t-test [33]. Regions in the volume that show a significant difference between conditions are assumed to be involved in processing the stimulus of interest.

The active windows were compared against the passive pre-stimulus baseline using two separate bandpass filters. The analysis focused on theta (4–8 Hz) and gamma (60–90 Hz) activity and these bandwidths were chosen with reference to Osipova et al [21] in conjunction with the sensor-space analysis. Statistical maps of voxel-wise t-values for each condition, time interval, and filter were created for each participant, which showed significant changes in power in each active window against the passive baseline window. Group statistics were then carried out on the individual t-maps to establish consistent activations at the group level. The resulting group maps were co-registered on to a standardised brain (Montreal Neurological Institute) and thus all reported co-ordinates are in MNI space. The chosen statistical method was non-parametric permutation testing [34]. The data were thresholded in a non-parametric, data-driven permutation scheme consisting of 10000 iterations. This method overcomes the problems faced by increased familywise error rates that result from multiple comparisons [35], [36], [37]. All reported beamforming results are group analyses, and are reported at a corrected significance threshold of *p*<.05.

### Estimate of lateralisation

The group-level beamformer analysis was used to perform a volumetric analysis of cortical regions which showed a stimulus-related change in activity relative to baseline. These primary results establish that the frontal cortex shows increases in theta activity during both task (encoding/retrieval) and for both stimuli (verbal/non-verbal). In order to fully characterise the nature of the lateralisation of the response, a secondary region-of-interest analysis was performed.

For this analysis, the theta-band beamformer analysis was re-run, but only grid points falling within left and right frontal cortices were included in the analysis. This analysis was performed in standard-space, but thresholds were calculated individually for each participant using non-parametric statistics and a label exchange of active and passive epochs. The calculated threshold (p<0.05) was used as a critical value and the number of significant grid locations in each hemisphere was summed for each stimulus-task combination (verbal-encoding, verbal-retrieval, non-verbal-encoding, non-verbal-retrieval). This analysis was performed separately for the early and late analysis windows already described.

## Results

### Behavioural

Participants correctly identified 87% of the target stimuli (Doors 89%, Names 86%), and 88% of the foil stimuli (Doors 86%, Names 90%). There was no significant difference in the amount of correctly identified targets between Doors (M = 85.12, SE  = 1.88), and Names (M = 82.31, SE = 2.68, t(15)  = 1.665, *p*>.05). Paired sample t-tests showed that there was no significant difference in reaction times between correct items for Doors (M = 553.71, SE  = 39.05), and Names (M  = 501.94, SE = 29.66, t(15)  = 1.704, *p*>.05). There was also no significant difference in reaction times to incorrect items for Doors (M = 599.18, SE = 48.35), and Names (M = 614.02, SE = 47.82, t(11)  = -.247, *p*>.05).

### Sensor Space Analysis

**Figure 4:**
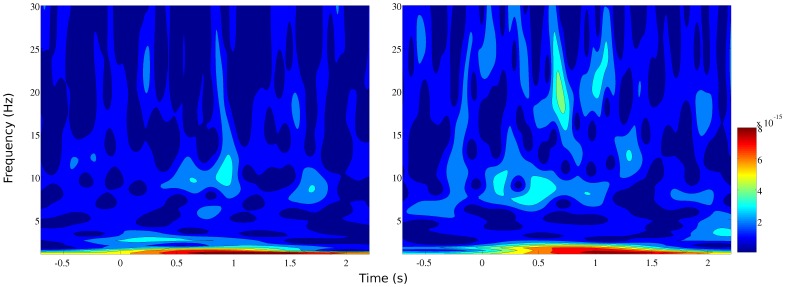
Event-related fields from the sensor-space analysis are shown for a representative subject. The left-hand panel shows the response to Doors stimuli and the right-side to Name stimuli.

Individual trials were observed on the sensors in order to reject trials containing biological or environmental noise. The average number of epochs analysed in each condition was 75 (range = 56–89). At this stage of visualisation, it was clear that trials were not overtly affected by eye movements and blinks. Figure 4 shows event-related time-frequency plots for 1–30 Hz for a group of 23 channels covering the anterior portion of the sensor array. Inspection of figure 4 confirms that there are increases in oscillatory activity post-stimulus onset in the theta-band range and this activity is comparable across both verbal and non-verbal stimulus sets. The event-related gamma activity does not show any clear deviation from baseline. This may be due to the fact that frontal gamma activity is non-phase-locked rather than phase-locked.

### Source Space Analysis

Activation within the gamma frequency band was widespread, showing main peaks of activation bilaterally within the occipital lobes, with activation spreading to the parietal and temporal lobes in all four conditions. This profile of activity in visual region in the gamma band was predicted based on Osipova et al [21]. However, the focus for the present study was the PFC and there was no significant changes in gamma band activity in the frontal cortex.

Activation within the theta frequency band was seen bilaterally in occipital areas during the first 200ms, after which the activation was predominately located within the frontal lobes. The remainder of the results will focus on data within the theta frequency band in the PFC, as the main aim of the paper is to characterise modulations in oscillatory activity in the frontal areas rather than changes in visual cortex. All reported activations were statistically significant (alpha = 0.05 corrected).

### Encoding: Doors

**Figure 5:**
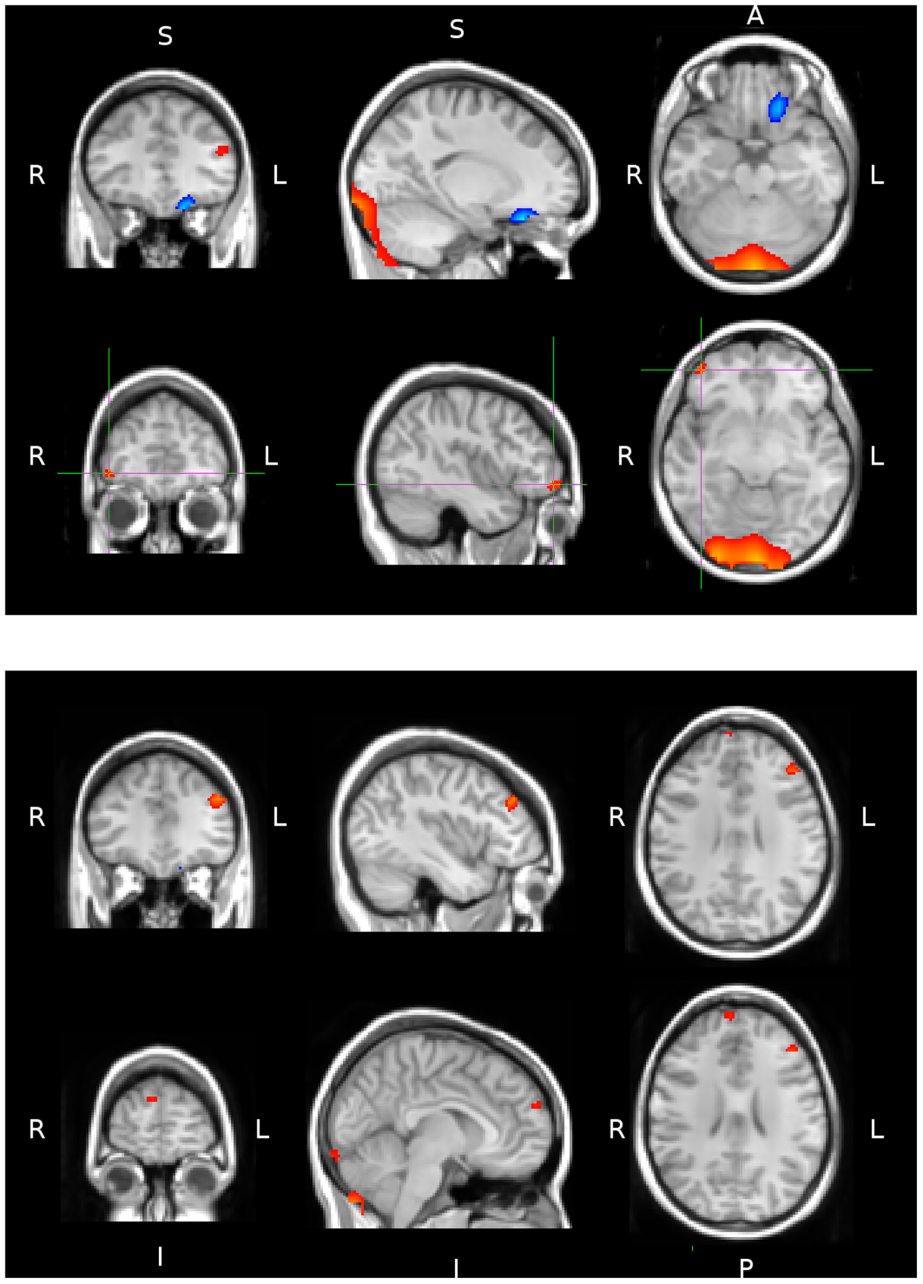
Regions identified as showing significantly greater activity in the theta band during encoding compared to baseline are shown as masks overlaid on a standard-space brain in radiological convention. The masks in blue and red show the activity for Name and Door stimuli respectively. The upper panel shows significant activity during the early analysis window centred at two locations (maximum t-value of 7.48 and 10.11 for Names and Doors respectively). The lower panel shows significant activity for the later analysis window (maximum t-value of 5.75 and 7.42 for the Names and Doors respectively).

The upper panel and lower panels of figure 5 show in red/yellow the significant activity found for the early and late analysis windows respectively. Significant activation was displayed in the right ventrolateral prefrontal cortex (VLPFC) and in the left dorsolateral prefrontal cortex (DLPFC) during the early encoding phase. The second analysis window (in the lower panel) shows that the left DLPFC activity is sustained and this is accompanied by activity in the right dorsomedial prefrontal cortex (DMPFC).

### Encoding: Names

The only significant activation for the name stimuli during encoding was in the left ventromedial prefrontal cortex (VMPFC). This activity was found during the first analysis window and was severely diminished in the second window. A comparison of activation profiles for the two stimulus types confirms that the doors prompted more activity relative to baseline compared to names. Whilst activity in response to the doors was present in both analysis windows the Names stimuli only revealed early activity.

### Retrieval: Doors

**Figure 6:**
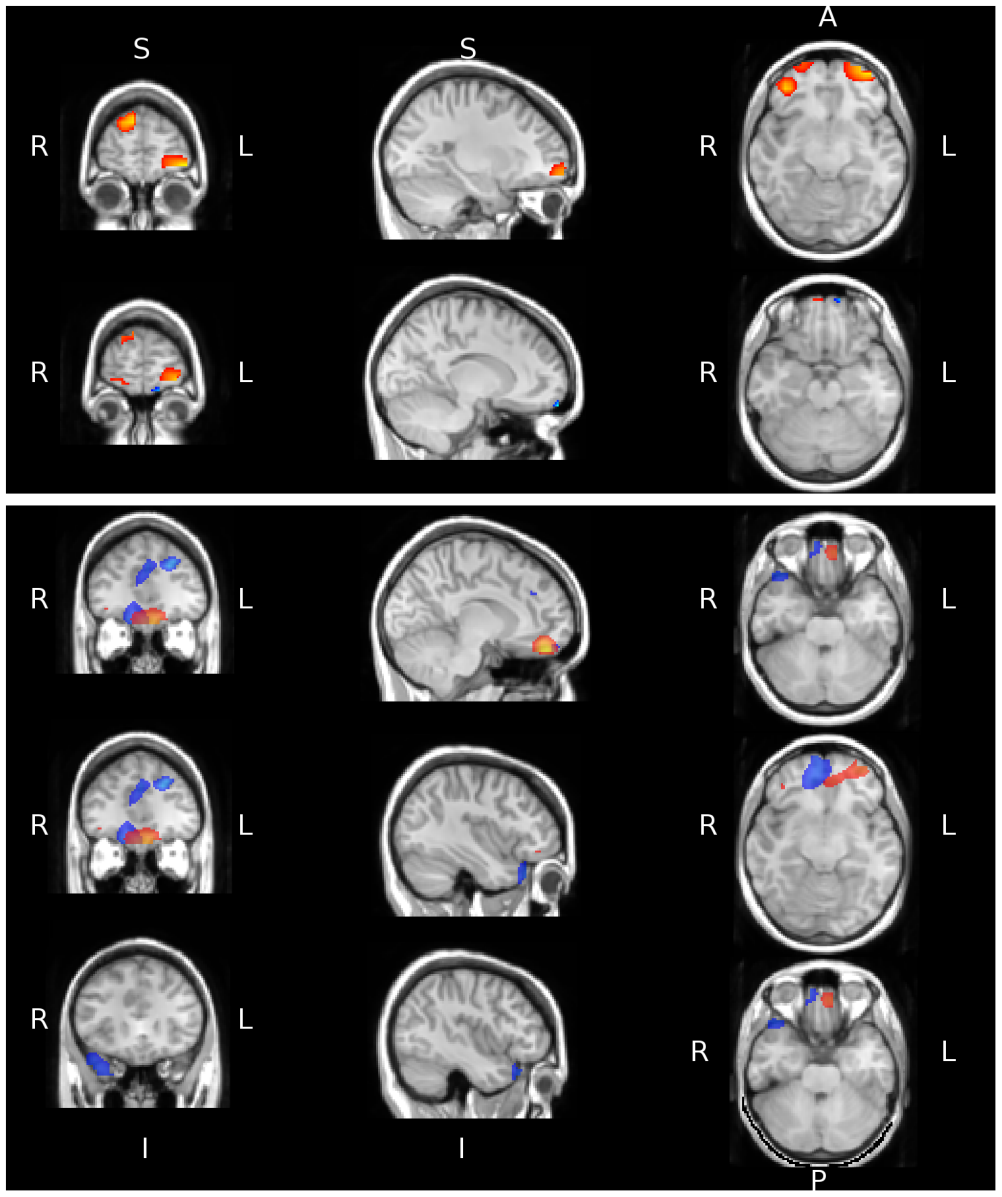
Regions identified as showing significantly greater activity in the theta band during retrieval compared to baseline are shown as masks overlaid on a standard-space brain in radiological convention. The masks in blue and red show the activity for Name and Door stimuli respectively. The upper panel shows significant activity during the early analysis window centred at two locations (maximum t-value of 5.82 and 6.66 for Names and Doors respectively). The lower panel shows significant activity for the later analysis window (maximum t-value of 7.5 and 7.38 for the Names and Doors respectively).

Figure 6 shows three clear nodes of activity in the early analysis window (shown in the upper panel of the plot. These regions are both left and right anterior prefrontal (APFC) cortices, combined with the right DMPFC. The activity in the later window is confined to the bilateral APFC, although this activity is strongest in the left hemisphere. The results suggest that, as was seen with the analysis of encoding trials, the activity in response to the non-verbal doors stimulus may occur earlier than for the name stimuli.

### Retrieval Names

The first analysis window shows little significant activity, with only a small region in the left APFC reaching significance. The second analysis window sees more regions recruited in response to the task of retrieving information related to the verbal name stimuli. The activity is in the left DMPFC and the right APFC. There is also significant activity in the right temporal pole region.

### Laterality of the response

**Figure 7:**
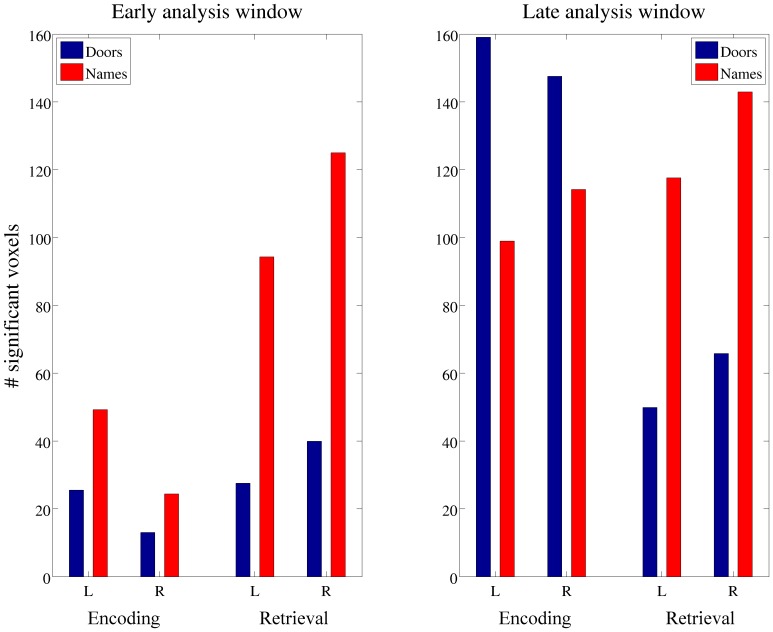
The number of significantly active voxels present in left and right frontal cortices are shown for each stimulus-task pairing, averaged across participants. The left-hand panel shows the early analysis window and the right-hand panel the later analysis window.

Each participant yielded 8 values from the secondary region-of-interest analysis for both the early and the late analysis windows. These values, averaged across participants, are shown in figure 7 and provide an overall characterisation of the laterality of the response. The early window indicates that there was greater significant activity for retrieval compared to encoding. The *Names* and *Doors* stimuli both show subtle differences between left and right hemispheres and a repeated-measures 2×2×2 ANOVA confirmed there was a significant main effect of task (retrieval > encoding), *F*(1,15), 5.664, *p*<0.05), no main effect of stimulus (verbal/non-verbal, *F*(1,15) 0.367, ns) or hemisphere (left/right, *F*(1,15), 0.444, ns).

The data from the late analysis window were also subjected to a repeated-measures 2×2×2 ANOVA which confirmed there was no significant main effect of task ( *F*(1, 15) = 0.348, ns) of stimulus ( *F*(1, 15) = 0.003, ns) or of hemisphere ( *F*(1, 15) = 0.952, ns). One of the most striking aspects of these data is the difference observed when comparing the early and late analysis windows and this clearly indicates that the network of activity is dynamic and changes as a function of time. For the statistical analyses performed, time (early vs late) was not included as a factor in the ANOVA and the two were evaluated independently. The time-windows were selected based on the sensor-space ERFs and it is possible that these are more appropriate for some task-stimuli-window combinations than others. Furthermore the region-of-interest voxel-based count statistic is a crude metric which reduces the rich neuronal signature captured in a very blunt manner. What is required to understand the dynamics of the response more fully is a study which utilises the full time course of the response in a statistic. Due to the slow duty cycle of theta rhythms the current study is not optimised to do this. The differential activity that is clearly observed across the two windows suggests such a study, optimised for this specific question, would be of value.

## Discussion

Distinct patterns of neural activity in the PFC for successful encoding and retrieval in verbal and non-verbal memory were identified using the Doors and People Test of recognition memory. The two main points demonstrated by the study are that a standard, neuropsychological assessment can be successfully adapted to allow sufficient signal-to-noise and task related activity to perform a meaningful neuroimaging study. Secondly, the experiment described yields clear regions of importance for the verbal and non-verbal memory tasks performed. These tasks recruit dorsal/ventral and lateral/medial regions in a task and stimulus specific manner.

The general predictions based on the literature stated that verbal activity would maximally stimulate the left prefrontal cortex whereas non-verbal stimuli would activate the right frontal regions more strongly. In general, the whole-head volumetric analyses support this prediction although in addition to these gross patterns of activity there was clearly bilateral neuronal activity for some tasks. The other general prediction was that encoding would be lateralised more strongly to the left hemisphere, and retrieval to the right hemisphere. Although the activation profiles show a dominance towards this pattern of lateralisation, the network of activity is clearly more distributed and bilateral than a simple prediction of laterality would allow. Furthermore the region-of-interest analysis, which statistically evaluates the number of significant voxels in each frontal region, suggests remains statistically underpowered and thus definitive inferences are difficult to make. The region-of-interest approach is necessarily crude, as it reduces a complex, dynamic signal into a single “score” for each hemisphere, and is not sensitive to the relative contribution of the sub-regions within a hemisphere. However, if these data are taken as a further descriptor of the whole-head analysis presented, it remains clear that nodes of a frontal network of memory can be elucidated in MEG and the pattern of this network is more complex than the literature currently acknowledges. These regions, and the relative contribution of smaller sub-regions to the processes of encoding and retrieval are suitable for further investigation using MEG as an investigative tool.

### Encoding

During the encoding phase there was material-specific profiles of activation, with non-verbal stimuli recruiting the left dorsolateral prefrontal cortex across both analysis windows. This region showed the strongest stimulus-driven change of all the frontal in response to non-verbal encoding. This DLPFC activity was accompanied by right-sided ventrolateral activity in the early window and right-sided ventromedial activity in the second analysis window. In contrast to this activation profile, the verbal encoding task showed only left-sided ventromedial prefrontal activity and this was only present for the first analysis window, which suggests a difference in the timing of the responses between verbal and non-verbal stimuli with verbal stimuli activating the nodes of the frontal network more quickly than non-verbal stimuli. The VLPFC was found to show a level of stimulus-specific laterality, with non-verbal stimuli activating an area in the right VMPFC, and verbal stimuli activating an area in the left VMPFC during encoding.

Previous studies reporting material-specific laterality for the encoding of episodic memory found the activations in lateral regions of the PFC [5], [7], [8], [13]. The current study demonstrated medial activations of material-specific laterality, located within the left VMPFC for verbal material, and right VMPFC for non-verbal. Our findings support the hypothesis that there is a material-specific laterality in the PFC during encoding.

### Retrieval

Both verbal and non-verbal stimuli significantly activate the anterior prefrontal cortices, and this activity shows a stimulus dependent pattern. The verbal stimuli are restricted to the right APFC and the non-verbal stimuli, although bilateral, predominantly activates the left APFC. The DMPFC is also recruited by both stimulus types during recognition, although converse to the APFC activity this shows right-sided activity to non-verbal stimuli and left-sided activity to verbal stimuli. Recognition of non-verbal stimuli activates the frontal network in the first analysis window whereas the verbal stimuli do not, this is in contrast to the temporal profile seen during encoding where the non-verbal stimuli appear to activate the frontal network later than the verbal stimuli.

Interestingly there is clear activation of the right-sided anterior temporal region during successful recognition of the verbal material. The anterior temporal lobe has been implicated in verbal recall tasks [38]. Many studies suggest that the left anterior temporal regions are important in verbal memory tasks, however others consider a bilateral representation to be a more accurate account of the neural encoding. It is certainly encouraging that the verbal recognition paradigm described in the current work was able to elicit stimulus-related activity in these anterior temporal regions.

The material-specific model would predict that recognition of verbal material would be lateralised to the left PFC, and non-verbal to the right PFC [8], [13]. Although the verbal stimuli activate predominantly left-sided PFC regions and the non-verbal right-sided, the APFC is bilaterally recruited for both stimulus types. Thus, although there are distinct patterns of activity dependent upon material type, the lateralisation is not in line with the material-specific model.

These patterns of activity result from the whole-head, volumetric analysis of activity and thus is a clear demonstration that MEG in combination with a spatial filtering analysis is an appropriate methodology with which to investigate neural processes of memory. Although the region-of-interest analysis provides some further description of the gross lateralisation of the response, what is needed are further studies which focus on better delineating the specific roles of the different frontal regions (APFC, DMPFC) using paradigms potentially better suited to such an aim, such as tasks with different attentional demands, analysing trials where retrieval was unsuccessful and directly contrasting active periods rather than using a shared passive baseline.

### Timing of the response

The use of MEG in the present study provided the opportunity to characterize for the first time the temporal components of PFC activity associated with episodic memory: most previous research has been carried out using fMRI and PET [3], [4], [5], [7], [8], [13] which are temporally insensitive. The use of an early and late analysis window demonstrated that for encoding the processing of verbal stimuli occurs early, with little sustained or late activity. When encoding non-verbal stimuli the left DLPFC was activated early and this region was also found to be present in the later analysis window. The second analysis window then also saw activity in the right hemisphere in the VLPFC and DMPFC. Therefore although the results clearly to not wholly support either the HERA or the material-specific model, it may be that these models are not designed adequately to cope with a dynamic system. The prefrontal cortex has clear nodes that are implicated in the successful encoding and retrieval of stimuli and it may be that any model of lateralisation must also consider the time-scale on which this laterality may occur for. One hemisphere may be dominant, and may contribute the early components of the response before other, cross-hemisphere regions are then implicated. It may also be necessary to expand the concept of laterality to distinguish between ventral and dorsal processing streams.

### Summary of Lateralisation

The key aim of the paper is to focus on the question of lateralisation of function (encoding vs retrieval) or stimulus (verbal vs non-verbal) set in the prefrontal cortex. The initial volumetric analysis presented in the results section indicates that the question of laterality is not straight forward, and it also changes as a function of time.

The secondary, region-of-interest analysis aimed to further explore the issue of laterality by using the number of significant voxels in each hemisphere as a metric. The bar plots presented suggest that there may well be differential hemispheric effects. For example in the late analysis window, for both encoding and retrieval, for both verbal and non-verbal stimuli, the right hemisphere appears to be dominant. Whereas in the early window, for the retrieval task, there is potentially an interaction between hemisphere and stimulus type. Although it may be tempting to draw such inferences from the presented data, the statistical analyses indicate that there is no basis upon which to do so, and the cross-participant variability is too high to perform a robust analysis. Furthermore, using the number of significant voxels as a metric of laterality is not ideal due to the inherent smoothness of beamformer images. For example it is likely to be crucial to consider not only the number of significant voxels but also the extent of a significant cluster and magnitude of this activity.

Despite these caveats, and the non-significant statistical effect of hemisphere, the information presented acts as a descriptive statistic to more fully describe the changes in oscillatory activity induced by the tasks used. Inspection of figure 7 reveals two key points that are crucial for further behavioural and neuroimaging studies in this area. The first is that the pattern of activity does not clearly fit with either of the two theories currently postulated regarding how hemispheric differences for memory function may manifest themselves, and the data analysed in this experiment suggest both accounts need further refinement. Secondly, the pattern of activity changes as a function of time, and it is crucial that future studies consider the frontal network of memory function not as a stationary network that is stable over time, but one consisting of different nodes which have different temporal characteristics.

### General Conclusions

These findings provide functional neuroimaging evidence that the verbal and non-verbal recognition components of the Doors and People test recruit two anatomically distinct memory systems, consistent with evidence from neuropsychological studies [10].

It is clear that further research is needed into the different temporal profiles of verbal and non-verbal episodic memory in the PFC. Specifically, replication of the distinct temporal activation differences in the right APFC and DMPFC, which are dependent on material, is needed. It seems that many researchers have been quick to completely dismiss the HERA model in light of growing evidence from fMRI for material-specific laterality, however, we have shown using MEG that there may still be a place for the HERA model, although the PFC appears to operate at a level of complexity that is beyond the explanation of a single model of laterality.
